# Impact of the biopsy forceps size on histological analysis and performances of the histological scoring systems

**DOI:** 10.1038/s41598-022-09704-w

**Published:** 2022-04-05

**Authors:** Elettra Bianchi, Aurélie Najm, Sophie Vanbelle, Benoit Le Goff, Eugène Mutijima, Marie-Joëlle Kaiser, Michel Malaise, Jean-Philippe Hauzeur

**Affiliations:** 1 Department of Pathology, CHU Sart Tilman, University of Liège, Liège, Belgium; 2 Department of Rheumatology, Centre Hospitalier Universitaire de Nantes, Pays de La Loire, Nantes, France; 3 Institute of Infection, Immunity and Inflammation, College of Medical Veterinary and Life Sciences, University of Glasgow, Glasgow, UK; 4 Methodology and Statistics, CAPHRI, Maastricht University, Maastricht, The Netherlands; 5 Department of Rheumatology, CHU Sart Tilman, University of Liège, Liège, Belgium

**Keywords:** Diseases, Rheumatic diseases

## Abstract

To improve the reliability of the quantitative scorings of the synovial biopsies, we evaluate whether diameter of arthroscopic forceps influences histological quality of synovial tissue and/or histological scores and we compare the intra- and inter-observer performances of the main histological scoring systems. Synovial biopsies were retrieved in the same part of the joint using 1, 2 and 4 mm diameters grasping forceps. After standard staining and immunohistochemistry with anti-CD68 antibody, slides were scored blindly by 2 independent experienced operators for tissue quality and with Krenn score, de Bois-Tak score and CD68 semi-quantitative score. Four samples did not pass quality control. No difference other than a higher number of vessels in the 4 mm versus 2 mm forceps (*p* = 0.01) was found among the 3 groups. CD68 score was significantly higher in the 2 versus 4 mm forceps (*p* = 0.009). So we concluded that only vessels quantification and CD68 semi-quantitative score seemed affected by the forceps size. The intra-reader agreement was variable across observers and features: 0.78 (0.66–0.87) for the Krenn scoring system, 0.89 (0.78–0.97) for the de Bois-Tak score and 0.93 (0.81–1.00) for the CD68 score. Interobserver reliabilities of Krenn score, de Bois-Tak score and CD68 scores were satisfactory: 0.95 (0.92–0.99) for Krenn, 0.98 (0.96–0.99) for de Bois-Tak and 0.80 (0.71–0.89) for CD68.

## Introduction

Synovial tissue analysis has been extensively studied over the past decade for both clinical and research purposes, especially for diagnosis or therapeutic evaluation, in Rheumatoid Arthritis (RA) and other rheumatic and musculoskeletal diseases (RMDs)^1^. Different techniques have been described in order to retrieve synovial tissue: blind punch biopsy techniques, arthroscopic biopsy with 1, 2. or 4 mm diameter forceps, Tru-Cut system or grasping forceps guided by ultrasonography (US)^2^. As the biopsy forceps diameter varies from 1 to 4 mm depending on the technique, the mean size of tissue retrieved supposedly varies accordingly. Moreover, different techniques have been described with different success rates in the literature. For example, Polley et al., with a 5 mm forceps, reported 86.2% adequate synovial tissue; in 3% specimens were not considered satisfactory for histological diagnosis because of the absence of lining cells or because of insufficient tissue and in 10.8% no synovial membrane was obtained^3^. Parker et al., using a 1.4 mm forceps, reported unusable biopsies in 4%^4^. Koski reported successful biopsy in 89% of the cases using a 2.7 mm forceps under US control, a^5^. Several groups reported various success rate using semi-automatic guillotines needles under US guidance of 81.6% in the clinical setting^2^ and from to 88% to 93% for synovial biopsies in research^6,7^.

Of interest, the biopsy technique appears to influence synovial tissue yield, quality^8^ and tolerability. However, among the same technique, the influence on the forceps type and size on tissue quality and analysis remains unclear.

On the other hand, the histological analysis of synovial tissue is used in many applications related to research mainly and especially evaluate response to therapy. Hematoxylin and eosin (H&E) is the most common staining performed and different scoring systems exist for synovial inflammation assessment. The first has been described by Krenn et al.^9,10^ and the second by de Bois^11^ and Tak et al.^12,13^. Immunohistological analysis are frequently performed usually using semi-quantitative or quantitative scoring systems. An update of Krenn score (KS) associating H&E and immunohistochemistry (CD3, CD20, CD68, Ki67 and CD31) has been recently proposed, improving the diagnostic performances of Krenn et al. score for inflammatory arthritis recognition^14^. In addition, the cell infiltrate can be defined as types which have been associated with difference disease phenotypes or prognosis^15^. While different scores exist for inflammation grading, their performances in terms of reproducibility or intra-inter observer reliability have been not enough described in the literature and need to be assessed. Moreover, the correlation between the different scores is not well established.

In an objective of contributing to standardization of the procedures in the field of synovial biopsies, it was felt important to address the knowledge gap pertaining to the influence of the type of instrumentation used in arthroscopy guided biopsies. Additionally, there is currently no preferred scoring system in synovial tissue histological analysis, and their reliability across different readers needs more research.

Therefore, the aims of this work were a) to evaluate the histological differences from biopsies retrieved from the same joint using different size of grasping forceps (1, 2 and 4 mm) in patients with RMDs and b) assess the intra and inter reader reliability for different synovitis scoring systems.

## Results

### Demographic and clinical information

Twenty knees were biopsied from twenty patients: 11 RA (ACR criteria), 3 Osteoarthritis, 2 Psoriatic Arthritis (CASPAR criteria), 1 Spondyloarthritis (ESSG criteria), 1 Chondrocalcinosis, 1 Osteonecrosis and 1 Diffuse Villonodular Synovitis. The mean age was 58.1 Years (39–78), the gender was male in 10 cases, and the joint was, in 16 cases, the right knee.

### Comparison of scores according to the biopsy forceps diameter

Forty nine biopsies were analyzed. Four samples did not pass the quality control as the lining layers could not be identified (1 mm biopsy forceps n = 1, 2 mm biopsy forceps n = 3) Among the forty five available biopsies, nine (20%) were obtained by 1 mm forceps diameter (4 RA, 2 Osteoarthritis, 1 Psoriatic arthritis, 1 Spondyloarthritis and 1 Diffuse Villonodular Synovitis), 18 (40%) by 2 mm forceps diameter (10 RA, 2 Osteoarthritis, 2 Psoriatic arthritis, 1 Spondyloarthritis, 1 Chondrocalcinosis, 1 Osteonecrosis and 1 Diffuse Villonodular Synovitis) and 18 (40%) of 4 mm forceps diameter (10 RA, 3 Osteoarthritis, 2 Psoriatic arthritis, 1 Spondyloarthritis, 1 Chondrocalcinosis and 1 Osteonecrosis). A comparison between the different sizes of 4 versus 2 versus 1 mm from the same joint was available in 7 cases, 4 versus 2 in 17 cases, 4 versus 1 mm in 8 cases, and 2 versus 1 mm in 8 cases.

Table 1 summarizes the results of the scoring discrepancies between the sizes of 1 vs 2, 1 vs 4 and 2 vs 4. No statistical difference was found among the 3 groups (1 mm, 2 mm and 4 mm forceps) for the total KS or the subscales, and for the DTS, except for the number of vessels which was higher in the 4 mm versus 2 mm forceps (*p* = 0.01). On the opposite, CD68 SQS was significantly higher in the 2 mm forceps versus 4 mm (*p* = 0.009).

**Table 1:** intra-rater agreements according to size of biopsy forceps.

A. Krenn scoring system: Medians and *p* values						
Sizes	Median			*p* value*		
	1 (N = 9)	2 (N = 18)	4 (N = 18)	1 VS 2 (N = 8)	1 VS 4 (N = 8)	2 VS 4 (N = 17)
Synovial lining layers	2.00	1.00	1.88	0.46	0.87	0.55
stroma cells density	1.00	1.00	1.00	0.34	1.00	0.75
Inflammatory infiltrate	1.00	1.38	1.00	0.15	0.79	0.23
Total Krenn	4.00	4.25	4.00	0.67	1.00	0.78

B. de Bois – Tak scoring system and CD68 scoring system: medians and p-values						
Sizes	Median			*p* value*		
	1 (N = 9)	2 (N = 18)	4 (N = 18)	1 VS 2 (N = 8)	1 VS 4 (N = 8)	2 VS 4 (N = 17)
Synovial lining layers	1.50	0.97	0.76	0.59	0.45	0.61
PMN	0.00	0.00	0.00	1.00	0.36	0.18
Plasma Cells	0.50	1.00	0.88	0.16	0.67	0.36
Lymphocytes	0.00	0.88	0.50	0.13	0.27	1.00
Vessels	2.50	3.00	3.90	0.18	0.27	**0.01**
Total de Bois–Tak	5.50	6.25	6.00	0.24	0.67	0.83
CD68	2.00	1.50	1.50	0.26	0.92	**0.01**

*P value significant when less than 0.05/3 = 0.017.

Significant values are in bold.

### Intra and inter-reader reliability of the different scoring systems

In a first step, to study the intraobserver variability, 19 images were scanned and blindly scored by each observer at 2 different occasions 2 months apart.

Then, to study the interobserver variability, 57 biopsies were scanned and blindly scored by each observer. The mm sizes of the 57 biopsies were as follows: 9 at 1 mm, 24 at 2 mm and 24 at 4 mm. The results were entered onto a spreadsheet by each observer and directed to a central point to be analyzed.

Intra-observer agreement is provided for each subscale and for the total score in Table 2 for the KS, the DTS and the SQS. The intra-reader agreement was variable across observers and features. For the KS, the different between the test and retest scores was on average 1.2 points (95% CI: 0.79; 1.5) for observer A and 0.1 point (95% CI: 0; 0.26) for observer B. For observer A, the largest differences were observed in the scoring of stroma cell density. For the DTS, the difference between test and retest scores was on average 1.3 points (95%: 0.84; 1.7) for observer A and 0.3 point (0.1; 0.53) for B. For observer A, the largest differences in scoring were observed for lymphocyte density. The concordance correlation coefficient was 0.78 (0.66;0.87) for the KS, 0.89 (0.78;0.97) for the DTS and 0.93 (0.81–1.00) for the CD68 SQS.

**Table 2:** Subscales and total Krenn scoring system interobserver agreement and according to size of biopsy forceps.

	Biopsy size	N	% agreement	CCC (95% CI)
LB	1	9	1	1 (NA)
	2	24	95.2	0.98 (0.94 ;1.00)
	4	24	87.5	0.92 (0.83 ;1.00)
	Total	57	92.4	0.96 (0.92 ;1.00)
ST	1	9	0.89	0.93 (0.79 ;1.00)
	2	24	0.75	0.84 (0.71 ;0.96)
	4	24	0.83	0.86 (0.68 ;1.00)
	Total	57	0.81	0.86 (0.78 ;0.94)
IN	1	9	0.89	0.93 (0.79 ;1.00)
	2	24	0.92	0.96 (0.91 ;1.00)
	4	24	0.88	0.92 (0.83 ;1.00)
	Total	57	0.89	0.94 (0.89 ;0.99)
TK	1	9	0.75	0.71 (0.77 ;1.00)
	2	24	0.61	0.97 (0.94 ;1.00)
	4	24	0.63	0.91 (0.84 ;0.99)
	Total	57	0.64	0.95 (0.92 ;0.99)

LB = lining layers ; ST = stroma cells ; IN = inflammatory infiltrate ; TK = total Krenn ; NA = no available.

CCC = concordance correlation coefficient ; N = number of biopsies.

The interobserver results are presented for each subscale and for the total in Tables 2, 3, 4 concerning respectively the KS, the DTS and the CD68 SQS. The inter-rater agreement is generally good, with concordance correlation varying between 0.71 and 1 for KS and between 0.81 and 1 for DTS. The inter-observer agreement was 0.95 (0.92–0.99) for KS, 0.98 (0.96–0.99) for DTS and 0.80 (0.71–0.89) for CD68 SQS.

**Table 3:** Subscales and total De Bois-Tak scoring system inter-rater agreement and according to size of biopsy forceps.

	Biopsy size	N biopsies	% agreement	CCC	95% CI
SL	1	9	1	1	NA
	2	24	0.81	0.89	0.79–0.99
	4	24	0.83	0.87	0.73–1.00
	Total	57	0.84	0.91	0.84–0.98
PMN	1	9	0.89	0.88	0.62–1.00
	2	24	0.96	0.94	0.82–1.00
	4	24	1.00	1.00	NA
	Total	57	0.96	0.94	0.85–1.00
PC	1	9	0.89	0.91	0.74–1.00
	2	24	0.88	0.95	0.90–1.00
	4	24	0.96	0.97	0.92–1.00
	Total	57	0.91	0.97	0.93–1.00
LY	1	9	0.89	0.95	0.83–1.00
	2	24	0.88	0.95	0.90–1.00
	4	24	0.88	0.86	0.72–0.99
	Total	57	0.88	0.93	0.87–0.99
VX	1	9	0.78	0.95	0.88–1.02
	2	24	0.83	0.88	0.71–1.00
	4	24	0.92	0.93	0.83–1.03
	Total	57	0.86	0.93	0.86–1.00
LB	1	9	0.50	0.96	0.94–0.99
	2	24	0.62	0.98	0.96–0.99
	4	24	0.67	0.97	0.95–0.99
	Total	57	0.62	0.98	0.97–0.99

SL = lining layers ; PMN = Polymorphonuclear cells ; PC = plasma cells ; LY = lymphocytes ; VX = vessels ; LB = total de Bois-Tak ; NA = not available.

**Table 4:** Inter-rater agreement (CCC) for CD68 semi-quantitative score and according to size of biopsy forceps.

Size	N biopsies	% agreement	CCC	95% CI
1	9	0.50	0.71	0.56–0.87
2	24	0.74	0.74	0.59–0.90
4	24	0.79	0.85	0.71–0.99
Total	57	0.73	0.80	0.71–0.89

The correlation coefficient between the total scores of the two scoring systems is 0.78 (95%: 0.49–0.93), meaning that the two scales share 60% of variability.

## Discussion

The interest for histological quantitative analysis of the synovial tissue have been increasing over the past decade, more specifically for diagnosis and therapeutic assessment, especially in RA^1^. Interestingly, few works have aimed at looking into the impact of the biopsy forceps (type and size) on the procedures’ success rate or quality control of the biopsies. Furthermore, some author have mixed different techniques without mentioning possible sampling bias^16^. The process of obtaining synovial tissue involves different types of techniques, involving grasping forceps or biopsy needles from 1 to 4 mm diameters^2^. The quality of the synovial biopsy sample is an essential element for a reliable conclusion. This quality could, however, be compromised with the smallest biopsy forceps in term of morphology and/or cellular content. Interestingly, previous works reported significant correlations in biopsies performed in the same joint using different techniques: arthroscopy versus blind needle, especially for the mean lining cell depth and CD68+ lining layer infiltrate, and for the CD3+ infiltrate in the sublining^17^. However, the specific influence of biopsy forceps used were not described and their possible effect not discussed. Our study specifically focused on this aspect and we were able to describe tissue features according to the size of the forceps used.

Overall, we demonstrated a good histological quality of synovial biopsies obtained with 1, 2 and 4 mm grasping forceps. While all biopsies performed using a 4 mm forceps displayed a sufficient quality, only 1/9 (11.1%) 1 mm and 3/18 (16.7%) 2 mm were excluded because of the absence of visible lining layer.

To test the effect of the forceps size on histological outcome, the 2 most used semi-quantitative scoring systems were used. The scorings were done independently by 2 experts in synovial histology, as done in the previous publications^2,11–13,18–20^. We only found a statistical size effect when comparing 2 mm to 4 mm for the vessels quantification (*p* = 0.01) in the DTS and for the SQS (*p* = 0.009). For future studies including quantification of synovial samples, such findings suggest that the same forceps size should be used to avoid bias.

Moreover, scoring systems are multiple and their correlation and reliability has been assessed scarcely in the literature. In the present study, interobserver reliability appears satisfactory for the KS and DTS, for agreements values (0.95 for KS, 0.98 for DTS). Interestingly, some items such as the stroma cell density in the KS and scoring of lymphocyte density in the DTS however displayed a lower concordance. Our results are in line with previously published works for KS^9^. The overall intra-observer reproducibility rate (2 observers) was 72%, and the inter-observer 83%. In our study, the KS and DTS displayed a good correlation (Spearman 0.78). However, the differences of the items (KS unlike the DTS doesn’t score the vessels density nor, separately, the inflammatory cells) explain 60% of the total scores.

Regarding immunohistochemistry semi-quantitative scoring, we report a good inter and intra-observer reliability for CD68 scoring. Other immunostainings have to be studied, because they could need different scoring scales and could have other reproducibility levels^21^. Rooney et al., for a semiquantitative analysis, obtained ICC inter-observer agreement 0.84 for CD68+ and 0.80 for CD3+ ^22^.

Of interest, while some previously published work mentioned only minor differences of scoring between the 2 observers^12,13,19^ without provided percentage of agreement, Bresnihan et al. described an agreement of 0.86 between 3 observers for T lymphocytes quantification and 0.95 for vascularity quantification^23^. Intra-observer reliability is more rarely assessed or described in the literature. In our work, intraobserver reliability was very variable depending on parameters assessed, scores and observers, highlighting the variability in interpretation even for well-trained pathologists. However, although the reliability was overall, satisfactory, the sensitivity to change of all scores need to be better evaluated. In addition, agreement and guidance on which scoring system to be used for synovitis assessment would be interesting.

In this work we used the arthroscopic biopsy; ensuring to obtain biopsies from the same part of the knee and avoid bias due to the reported variability of the histological properties in the same joint^24^. It is possible that studies using different biopsy techniques such as notched needles like a Tru-Cut needle give other results because the synovial sampling process is quite different. Indeed, in comparison to grasping forceps, allowing a perpendicular grasp of the synovial tissue, the Tru-Cut retrieves the tissue tangentially.

Although some limitations should be considered on this study, especially regarding the different diagnoses along with small numbers of cases, our findings were significant and should be confirmed in larger cohorts.

In conclusion, although most of the tissue scoring were comparable regardless the forceps size, some parameters such as number of vessels quantification and CD68 semi-quantitative score seemed to be affected by the forceps size. The intra-reader agreement was variable across observers and features. In addition, interobserver reliability of both KS and DTS histological scores, along with SQS were satisfactory. Our results should be confirmed in other cohorts and with other immunohistological stainings than CD68.

## Material and method

The study was approved by the Ethics Committee of the Faculty of Medicine of Liège. All the patients signed n informed consent form. All methods were performed in accordance with the relevant guidelines and regulations.

The synovial biopsies were retrieved during diagnostic arthroscopy of the knee done under local anesthesia using lateral infrapatellar portal for the arthroscope (Storz Hopkins 30°, 4 mm diameter) and a lateral supra patellar portal for the grasping forceps^25^. Different grasping forceps were used having 1, 2 or 4 mm diameters. Biopsies were performed under arthroscopic vision, in the same section of the medial compartment of the knee and by the same operators (JPH and MJK).

Synovial tissue was fixed in 10% formaldehyde and then embedded in paraffin. Five µm-thick paraffin sections were cut. Finally, hematoxylin and eosin standard protocol stain was performed on deparaffinized slides. Immunohistochemistry with anti-CD68 antibody was also performed.

Immunohistochemistry was conducted on 4 µm-thick formalin-fixed, paraffin-embedded tissue sections mounted on SuperFrost Plus slides (Thermo Scientific) followed by drying at 60 °C for 120 min. Immunohistochemistry was performed on a Benchmark Ultra immunostainer (Ventana). Sections were stained with anti-CD68 (KP-1) antibody (Roche).

For each biopsy, sections were coded and high-powered field (HPF: 400X magnification) were randomly selected, digitalized, saved and analyzed: 5 HPF for the lining layer evaluation and 8 for the other items. For digital pictures the Case Viewer Native Window Application—3D HISTECH system was used^9^. Using this method, each observer received and scored the same digitalized histological images. This method is in accordance with the EULAR and OMERACT recommendations^17^.

Each slide was scored blindly by 2 independent experienced operators (EB and AN) from the patient name and the type of grasping forceps. A mean value was calculated for each item.

The histological quality of each biopsy was defined as a preserved morphology and a visible lining layer. Semi-quantitative analysis was performed using the following scoring systems: Krenn et al.^9,10^ and de Bois^11^ then by Tak et al.^12,13^ (summarized in supplementary Table 1S). The last scoring analysis for CD68+ macrophage infiltrate was the semi-quantitative (0–3) scoring system: 0 = no infiltrate, 1 = mild infiltrate, 2 = moderate infiltrate, 3 = severe infiltrate)^14^.

### Statistical analyses

Concerning the forceps size effect, the scores were summarized using the median and graphically displayed using dot plots. Because the sample size for the three biopsy sizes was different, groups were compared in pairs using a Wilcoxon signed rank test. Bonferroni correction was used to adjust for multiple comparisons. Since three biopsy sizes were compared, the significance level was fixed to 0.05/3 = 0.0167. Concerning the intraobserver and interobserver agreement, the levels were quantified in two ways: through the proportion of agreement (in percent) and through the concordance correlation coefficient (CCC)^26^. For sample sizes larger than 20, the CCC is equivalent to the intraclass correlation coefficient (ICC) based on two-way ANOVA. It quantifies how well subjects (images in this case) can be distinguished from each other in the population. Ninety five percent confidence intervals were constructed by accounting for the presence of repeated measurements on the same biopsy samples (e.g. different biopsy sizes of the same sample). The relationship between the total scores of the Krenn and the de Bois-Tak systems was summarized by Spearman correlation. Data analysis was conducted using R (version 3.2.5 for Windows). Missing values were not replaced.

### Ethics approval consent to participate

The study was approved by the Ethics Committee of the Faculty of Medicine of Liège.

## Supplementary Information

Supplementary Information.
