# Supplementary material for: Impact of the biopsy forceps size on histological analysis and performances of the histological scoring systems
Source: Sci Rep. 2022 Apr 5;12:5692. doi: 10.1038/s41598-022-09704-w (PMC8983678; doi:10.1038/s41598-022-09704-w)
Supplement: Supplementary file 1 — Supplementary Information. [file 41598_2022_9704_MOESM1_ESM.docx]

**Impact of the biopsy forceps size on histological analysis and performances of the histological scoring systems**

**Table S1** intra-rater agreements according to size of biopsy forceps

A. Krenn scoring system: Medians and p-values

|  | Median | | | p-value* | | |
| --- | --- | --- | --- | --- | --- | --- |
| Sizes | 1  (N=9) | 2  (N=18) | 4  (N=18) | 1 VS 2  (N=8) | 1 VS 4  (N=8) | 2 VS 4  (N=17) |
| Synovial lining layers | 2.00 | 1.00 | 1.88 | 0.46 | 0.87 | 0.55 |
| stroma cells density | 1.00 | 1.00 | 1.00 | 0.34 | 1.00 | 0.75 |
| Inflammatory infiltrate | 1.00 | 1.38 | 1.00 | 0.15 | 0.79 | 0.23 |
| Total Krenn | 4.00 | 4.25 | 4.00 | 0.67 | 1.00 | 0.78 |

* P-value significant when less than 0.05/3 = 0.017

Krenn scoring system: dot plots. Red point represent the mean.

| Synovial lining layers | Stroma cells density |
| --- | --- |
| 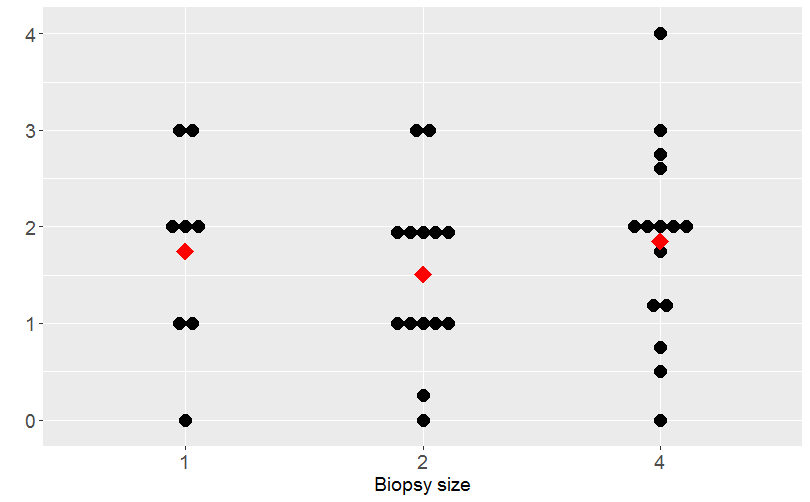 | 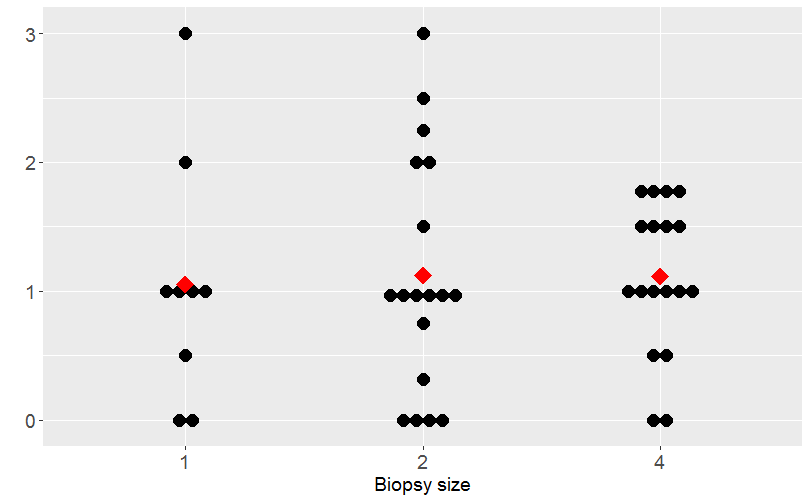 |
| Inflammatory infiltrate | Total Krenn |
| 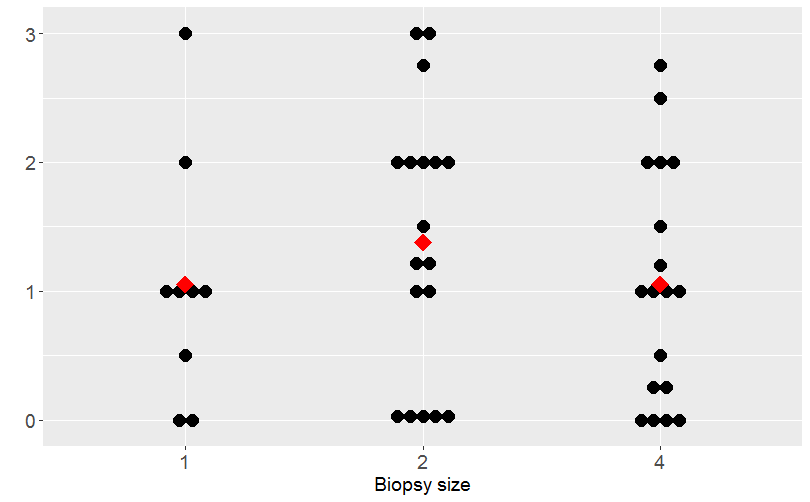 | 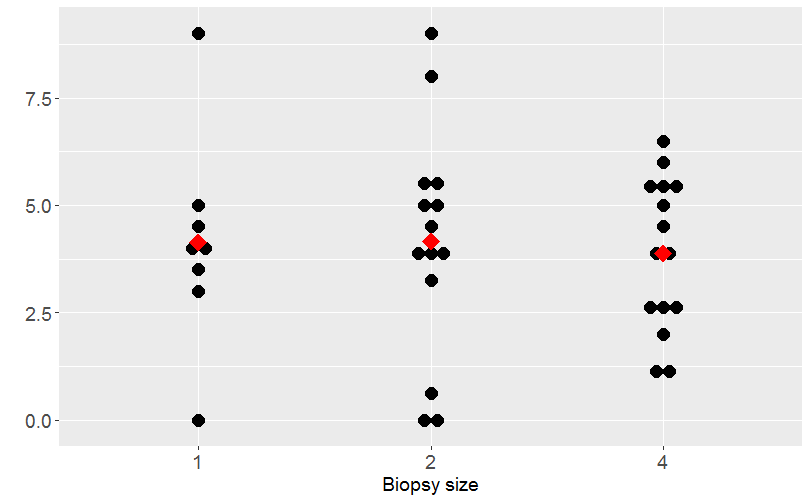 |

B. de Bois – Tak scoring system and CD68 scoring system: medians and p-values

|  | Median | | | p-value* | | |
| --- | --- | --- | --- | --- | --- | --- |
| Sizes | 1  (N=9) | 2  (N=18) | 4  (N=18) | 1 VS 2  (N=8) | 1 VS 4  (N=8) | 2 VS 4  (N=17) |
| Synovial lining layers | 1.50 | 0.97 | 0.76 | 0.59 | 0.45 | 0.61 |
| PMN | 0.00 | 0.00 | 0.00 | 1.00 | 0.36 | 0.18 |
| Plasma Cells | 0.50 | 1.00 | 0.88 | 0.16 | 0.67 | 0.36 |
| Lymphocytes | 0.00 | 0.88 | 0.50 | 0.13 | 0.27 | 1.00 |
| Vessels | 2.50 | 3.00 | 3.90 | 0.18 | 0.27 | **0.01** |
| Total de Bois - Tak | 5.50 | 6.25 | 6.00 | 0.24 | 0.67 | 0.83 |
| CD68 | 2.00 | 1.50 | 1.50 | 0.26 | 0.92 | **0.01** |

*P-value significant when less than 0.05/3 = 0.017

de Bois – Tak scoring system and CD68 scoring system: dot plots. Red point represent the mean.

| Synovial lining layers | PMN |
| --- | --- |
| 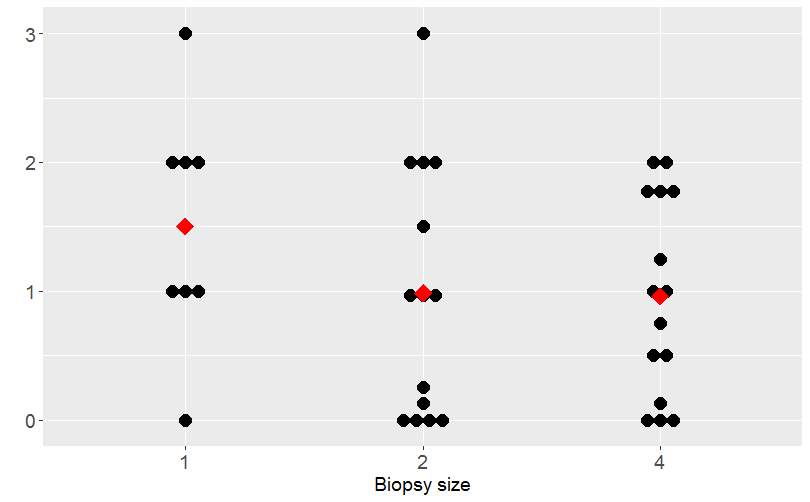 | 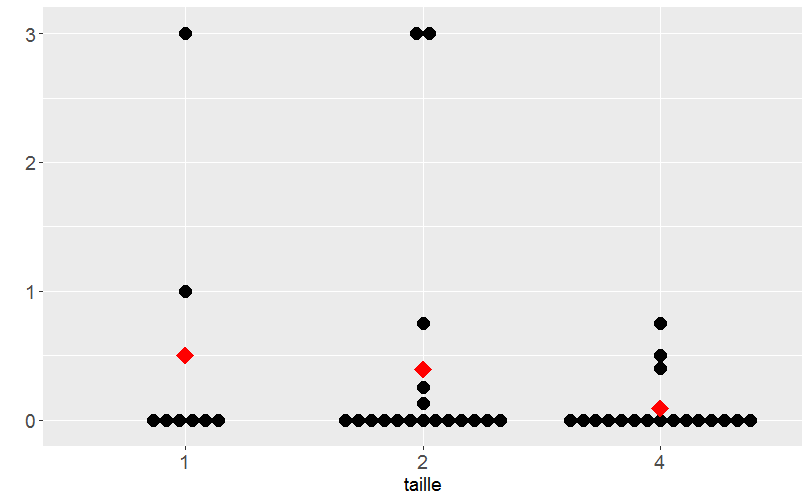 |
| Plasma Cells | Lymphocytes |
| 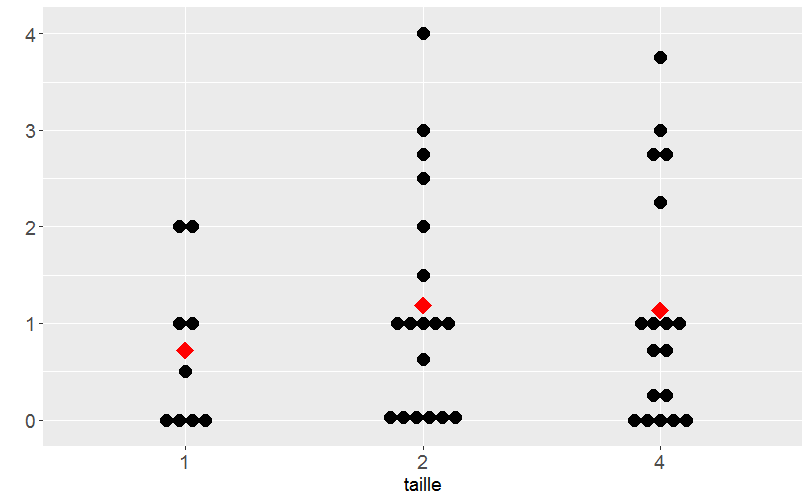 | 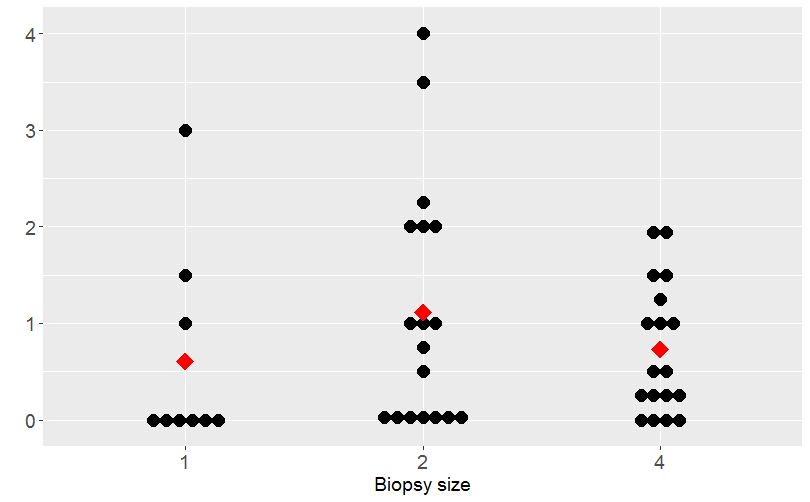 |
| Vessels | Total de Bois - Tak |
| 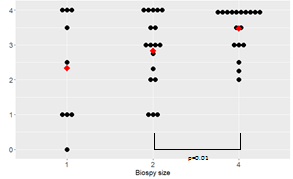 | 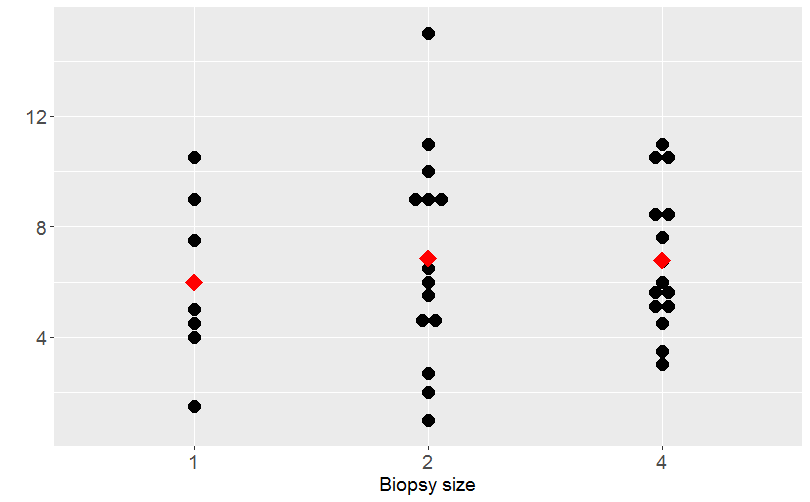 |
| CD68 |  |
| 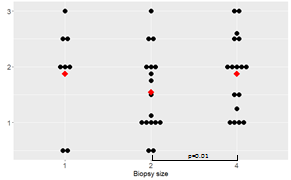 |  |
